# First Comprehensive *In Silico* Analysis of the Functional and Structural Consequences of SNPs in Human *GalNAc-T1* Gene

**DOI:** 10.1155/2014/904052

**Published:** 2014-03-04

**Authors:** Hussein Sheikh Ali Mohamoud, Muhammad Ramzan Manwar Hussain, Ashraf A. El-Harouni, Noor Ahmad Shaik, Zaheer Ulhaq Qasmi, Amir Feisal Merican, Mukhtiar Baig, Yasir Anwar, Hani Asfour, Nabeel Bondagji, Jumana Yousuf Al-Aama

**Affiliations:** ^1^Human Genetics Research Centre, Division of Biomedical Sciences (BMS), Saint George's University of London (SGUL), London, UK; ^2^Princess Al-Jawhara Al-Ibrahim Center of Excellence in Research of Hereditary Disorders, King Abdulaziz University, Jeddah, Saudi Arabia; ^3^Department of Genetic Medicine, Faculty of Medicine, King Abdulaziz University, Jeddah, Saudi Arabia; ^4^Dr. Panjwani Center for Molecular Medicine and Drug Research, International Center for Chemical and Biological Sciences, University of Karachi, Karachi 75270, Pakistan; ^5^Institute of Biological Sciences and Centre of Research for Computational Sciences and Informatics for Biology, Bioindustry, Environment, Agriculture and Healthcare (CRYSTAL, UM), University of Malaya, Kuala Lumpur, Malaysia; ^6^Faculty of Medicine, King Abdulaziz University, Rabigh, Saudi Arabia; ^7^Department of Biological Sciences, Faculty of Science, King Abdulaziz University, Jeddah, Saudi Arabia

## Abstract

*GalNAc-T1*, a key candidate of GalNac-transferases genes family that is involved in mucin-type *O*-linked glycosylation pathway, is expressed in most biological tissues and cell types. Despite the reported association of *GalNAc-T1* gene mutations with human disease susceptibility, the comprehensive computational analysis of coding, noncoding and regulatory SNPs, and their functional impacts on protein level, still remains unknown. Therefore, sequence- and structure-based computational tools were employed to screen the entire listed coding SNPs of *GalNAc-T1* gene in order to identify and characterize them. Our concordant *in silico* analysis by SIFT, PolyPhen-2, PANTHER-cSNP, and SNPeffect tools, identified the potential nsSNPs (S143P, G258V, and Y414D variants) from 18 nsSNPs of *GalNAc-T1*. Additionally, 2 regulatory SNPs (rs72964406 and #x26; rs34304568) were also identified in *GalNAc-T1* by using FastSNP tool. Using multiple computational approaches, we have systematically classified the functional mutations in regulatory and coding regions that can modify expression and function of *GalNAc-T1* enzyme. These genetic variants can further assist in better understanding the wide range of disease susceptibility associated with the mucin-based cell signalling and pathogenic binding, and may help to develop novel therapeutic elements for associated diseases.

## 1. Introduction

Glycosylation represents the common form of posttranslational modification that is critical for the stability, solubility, secretion, and interactions of proteins [1]. Of two glycosylation types (*N*- and *O*-linked), *O*-glycosylation is the most abundant glycosylation form of proteins expressed in a variety of secreted and membrane-bound mucins [2]. The *O*-linked glycosylation (also known as mucin type) is initiated in the Golgi compartment of human cells by the transfer of monosaccharide *N*-acetylgalactosamine (GalNAc) from UDP-GalNAc to the hydroxyl groups of Ser/Thr residues in core polypeptides by a large family (~20) of GalNAc-transferases (ppGalNac-Ts or GalNAc-Ts; E.C. 2.4.1.41) [3, 4]. These series of glycosyltransferases act together in elongating the *O*-linked glycan to produce a variety of densely *O*-glycosylated mucin glycoproteins. Mucin constitutes the principle component of mucus that defends the epithelial cell surfaces against infectious and environmental agents [5, 6]. Additionally, mucin-like glycans also serve as receptor-binding ligands during an inflammatory response [7].

The GalNAc-Ts (*GALNTs*) are classified into 27 family members based on their sequence and structural similarities. In total, 20 human GalNAc-Ts gene entries are made up-to-date. Most of the GalNAc-T genes, such as *GalNAc-T1*, *GalNAc-T3*, *GalNAc-T4*, *GalNAc-T5*, *GalNAc-T6*, *GalNAc-T7*, *GalNAc-T9*, *GalNAc-T12*, *GalNAc-T13*, and *GalNac-T14*, are important in determining the peptide and glycopeptide substrate specificities. Analysis of intron/exon positioning in human, fish, fly, and worm supports the model that GalNAc-Ts evolved from a common ancestral gene. Furthermore, several studies have provided the evidence that predicted orthologs of GalNAc-Ts genes in human, mouse, and fly represent true functional orthologs [4, 8, 9].

Over the past few years, there has been considerable interest in linking the genetic variations of GalNAc-Ts to disease susceptibility in humans. For example, rs17647532 in *GalNAc-T1* is known to influence the risk of epithelial ovarian cancers [10], rs142046356 in *GalNAc-T2* is described to play a role in elevating the HDLc levels in some families [11], rs6710518 in *GalNAc-T3 *is strongly associated with bone mineral density and fracture risk [12], and rs52822348 in *GalNAc-T4 *increases the risk of acute coronary disease [13]. Additionally, genome wide studies have also revealed the biochemically inactivating germ line and somatic mutations in *GalNAc-T12* genes [14].

In recent years, computational approaches have been extensively used to identify the impact of deleterious nsSNPs in candidate genes by using information such as conservation of sequences across species [15], structural attributes [16], and physicochemical properties of polypeptides [17, 18]. By adopting computational algorithms, some studies have successfully classified highly functional SNPs out of a huge pool of disease susceptible SNPs of *BRCA1 *gene, ATM gene [19], and *BRAF* gene [20] based on their structural and functional consequences. Despite the availability of convincing data indicating the wide involvement of *GalNAc-T1* gene mutations in human diseases, computational analysis of coding SNPs (nsSNPs and sSNPs in exonic regions) and noncoding SNPs (Intronic, exonic 5′, and 3′ UTR SNPs) in candidate GalNAc-Ts genes still remains unexplored.

**Figure 1:**
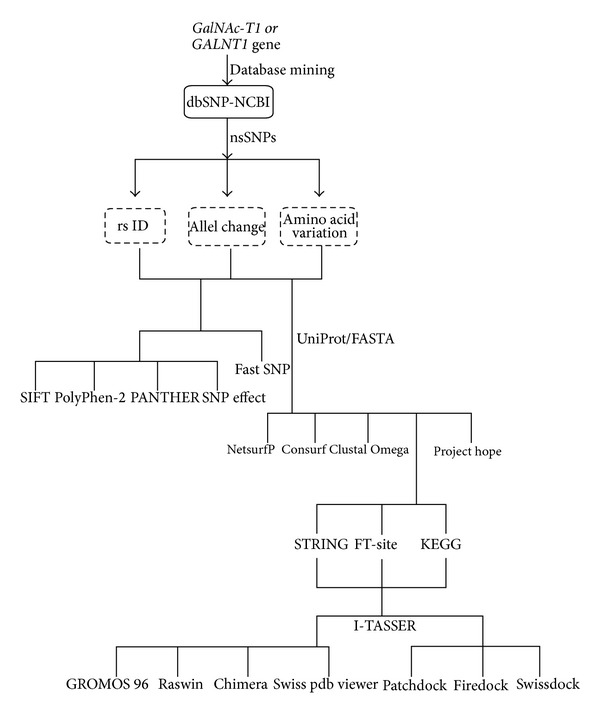
Schematic representation of computational tools for *in silico* analysis of *GalNAc-T1* gene.

Hence, in order to characterize the deleterious mutations in candidate GalNAc-Ts genes, the *GalNAc-T1* gene, which is known to be expressed at high levels and is found broadly among most tissues and cell types [21, 22], was chosen for *in silico* analysis in the current investigation. Our experimental strategy (Figure 1) involved (i) retrieval of SNPs in *GalNAc-T1* gene from public databases, (ii) classifying the deleterious nsSNPs to that of phenotypes, based on sequence and structure-based homology analyses and characterising the regulatory nsSNPs that alter the splicing and gene expression patterns, (iii) predicting the precise effects of amino acid substitutions on secondary structures by means of stability and solvent accessibility simulations studies, and (iv) analysing the impact of amino acid substitutions caused by nsSNPs on *GalNAc-T1* interactions with other proteins in a network. The *in silico* approaches adopted in this investigation offer advantages over the experimental based ones due to their convenience, reliability, speed, and of lower cost in order to locate the amino acid variants that regulate the function of GalNAc-T1 protein.

## 2. Methodology

### 2.1. SNP Data Mining

The data on the human *GalNAc-T1* (*GALNT1*) gene was collected from web-based data sources such as Online Mendelian Inheritance in Man (OMIM; http://www.ncbi.nlm.nih.gov/omim) and the National Center for Biological Information (http://www.ncbi.nlm.nih.gov/). Details of the SNPs (mRNA accession number, reference, and assay ID's) and protein sequence of *GalNAc-T1* gene were retrieved from the dbSNP-NCBI and SwissProt.

### 2.2. Analysis of Functional Consequences of nsSNPs by SIFT

Sorting Intolerant from Tolerant (SIFT; http://sift.jcvi.org/) predicts the tolerated and deleterious SNPs in order to identify the impact of amino acid substitution on phenotypic and functional changes of protein molecules. In the current investigation, the identification numbers (rs IDs) of each SNP of *GalNAc-T1 *gene obtained from NCBI were submitted as a query sequence to SIFT for homology searching. The SIFT value ≤ 0.05 indicates the deleterious effect of nonsynonymous variants on protein function [23, 24].

### 2.3. Simulation of Functional Consequences of nsSNPs by PolyPhen-2

Polymorphism Phenotyping-2 or PolyPhen-2 (http://genetics.bwh.harvard.edu/pph2/) is a probabilistic classifier which calculates functional significance of an allele-change by Naïve Bayes, a set of supervised learning algorithms. A mutation is evaluated qualitatively as probably damaging (probabilistic score > 0.85), possibly damaging (probabilistic score > 0.15), and benign (remaining), corresponding to the pairs of false positive rate (FPR) and true positive rate (TPR) thresholds, adjusted separately for HumDiv (10% & 18% FPR) and HumVar (19% & 40% FPR for probably and possibly damaging mutations, resp.). This tool was used to study the possible impacts of amino acid substitutions on the function of candidate GalNAc-T1 protein. Input options for PolyPhen-2 are comprised of UniProt accession number/FASTA sequence and detail of amino acids substitution [25, 26].

### 2.4. Characterization of Functional nsSNPs by PANTHER and Fathmm

The Protein ANalysis THrough Evolutionary Relationships (PANTHER; http://www.pantherdb.org/) classification system was used to characterize the functional nsSNPs in *GalNAc-T1* gene using HMM-based statistical modelling and evolutionary relationships in the protein family [27]. PANTHER-cSNP tools estimate the function of coding nsSNPs by calculating the subPSEC (substitution position-specific evolutionary conservation) score. The possible inputs to run the cSNP tools of PANTHER are the amino acid substitution and protein sequence. PANTHER subPSEC score ≤−3 classifies the amino acid substitution as deleterious or intolerant, whereas the score ≥−3 is predicted to be less deleterious [24].

The Functional Analysis through Hidden Markov Models (fathmm) was used to predict the functional, molecular, and phenotypic consequences of protein missense variants by combining sequence conservation within hidden Markov models (HMMs) [28]. User input for Fathmm server includes UniProt/ENSEMBLE ID with amino acid variation, prediction algorithm, and phenotypic associations.

### 2.5. Analysing the Molecular Phenotypic Effects of nsSNPs by SNPeffect

The SNPeffect database 4.0 (http://snpeffect.switchlab.org/) was used to predict the phenotypic impacts of nsSNPs of *GalNAc-T1 *gene. This database uses different tools such as TANGO (predict aggregation prone regions), WALTZ (simulate amyloidogenic regions), LIMBO (examine hsp70 chaperone binding sites), and FoldX (analyze the possible impact on protein stability) to annotate both noncoding and coding SNPs. Input options usually consist of a gene list and UniProt ID which are critical for a biological pathway or function. SNPeffect database mainly focuses on the molecular identification, categorization, and explanation of disease variants in the human proteome [29].

### 2.6. Identification of Functional SNPs in Conserved Regions by ConSurf

Using an empirical Bayesian inference, ConSurf web-server calculates the evolutionary conservation of amino acid substitution in proteins [30, 31]. After giving the FASTA sequence of *GalNAc-T1*, the conserved regions were predicted by means of conservation scores and colouring scheme, divided into a distinct scale of nine grades, starting from the most variable positions (grade 1) coloured turquoise, through intermediately conserved positions (grade 5) coloured white, to the most conserved positions (grade 9) coloured maroon [32].

### 2.7. Characterization of Functional SNPs in Regulatory Regions by FastSNP

Function analysis and selection tool for single nucleotide polymorphisms or FastSNP (http://FastSNP.ibms.sinica.edu.tw/) was used to analyse the SNPs located in regulatory regions of *GalNAc-T1* gene. This tool follows the decision tree principle to extract the functional information of SNPs from external web-servers and accordingly assign the risk rankings for SNPs from 1, 2, 3, 4, or 5 (which indicates the levels of very low, low, medium, high, and the very high functional impact, resp.). The input to query FastSNP is usually a gene symbol, SNP reference cluster ID (rs ID), or a chromosome position. After getting query, FastSNP provides two main options (query by candidate gene and query by SNP) to further generate the SNP function report against each SNP [33].

### 2.8. Analysis of SNP Effects on Surface and Solvent Accessibility of Protein by NetSurfP

Solvent accessibility or accessible surface area (ASA) of an amino acid helps in locating the potential active sites in a three-dimensional structure and in an extended tripeptide conformation of proteins (Shandar Ahmad et al. 2004). Amino acid FASTA sequence of *GalNAc-T1* was submitted to the NetSurfP (http://www.cbs.dtu.dk/services/NetSurfP/) server to predict its secondary structure, surface, and solvent accessibility of amino acids. This prediction method relies on the *Z*-score, which can predict the surfaces but not secondary structures of proteins. There are 3 subclasses defined for solvent accessibility of the amino acids; these include buried (low accessibility), partially buried (moderate accessibility), and exposed (high accessibility) [34]. Simulating the secondary structure of proteins is essential to analyze the relationship between amino acid sequence and protein structure. Hence, we simulated the secondary structure of GalNAc-T1 protein with I-TASSER (http://zhanglab.ccmb.med.umich.edu/I-TASSER) by excising continuous fragments from threading alignments and further refined them using replica-exchanged Monte-Carlo simulations. The quality of prediction models was reflected in the form of *c*-scores (−5 to 2). Secondary structure prediction and solvent accessibility analysis were an intermediate step prior to predicting the tertiary structure of GalNAc-T1 protein.

### 2.9. Modelling the Molecular Effects of nsSNP on Protein Structures

The nsSNPs can significantly change the stability of proteins. Consequently, in order to investigate the structural deviations and stability differences between native and mutant forms of *GalNAc-T1*, structural analysis was undertaken based on the results obtained from highest SIFT, PolyPhen-2, SNPeffect, and PANTHER scores. Prediction of the 3 dimensional model of GalNAc-T1 protein structure was done using the Universal Protein Resource (UniProt; http://www.uniprot.org/), I-TASSER (http://zhanglab.ccmb.med.umich.edu/I-TASSER/), and Multisource protein structure threading (MUSTER; http://zhanglab.ccmb.med.umich.edu/MUSTER/) web tools. However, structural visualization was performed by the SWISS-PDB viewer and Chimera [35, 36].

### 2.10. Analysis of Structural Specificity of Functional SNPs

To study the structural effects of mutations on GalNAc-T1 protein, Project Have yOur Protein Explained (HOPE: http://www.cmbi.ru.nl/hope/home), a unique web server, was used. To simulate structural features of mutations on native protein molecule, Project HOPE uses the 3D structures of the proteins that are available in Uniprot database and also in DAS prediction servers; however, the HOPE server can also build homology models independently, if necessary. The HOPE sever predicts the structural variation between native and mutant residues [37].

### 2.11. Prediction of Ligand Binding Sites on Unbound Protein Structures by FTSite

To solve the classic problem associated with the elucidation of protein structure-function relationship, protein engineering, and drug design, protein binding sites are considered as hot spot regions, especially for small ligand molecules. FTSite is an accurate computational method based on experimental evidence to determine the ligand binding sites with experimental accuracy of 94%. The input options contain job name, PDB ID or file, PDB chain IDs, and email (optional) to retrieve the binding sites/residues within the candidate unbound protein [38].

### 2.12. Predictions of Protein-Protein Interactions

Protein-protein interaction networks are important to unveil and annotate all functional interaction among cell proteins. For this current investigation, the online database resource “Search Tool for the Retrieval of Interacting proteins” (STRING; http://string-db.org/) was used. This provided unique coverage and ease of access to both experimental and theoretical interaction evidence of GalNac-T1. The input options for STRING database include protein name, protein sequence, and multiple sequences. STRING database is presently equipped with 5,214,234 proteins belonging to 1133 organisms [39]. To predict the functional networking of *GalNAc-T1* enzyme, we used KEGG (http://www.genome.jp/kegg/) PATHWAY and LIGAND and curated the data for molecular reaction and interaction networks, including metabolic pathways, regulatory pathways, and molecular complexes for biological systems.

### 2.13. Molecular Docking for Protein-Ligand Interaction by PatchDock and FireDock

To get the structural insight of unbound protein-ligand interaction, PatchDock and FireDock (Fast Interaction REfinement in molecular DOCKing)**  **were considered. PatchDock algorithm entails object recognition and image segmentation techniques to carry out rigid docking through a three-stage filtering process: (a) molecular shape representation, (b) surface patch matching, and (c) filtering/scoring. The FireDock method simultaneously targets the problem of flexibility and scoring of solutions to address the refinement problem of protein-protein docking solutions. After generating top 1000 models, the PatchDock output was redirected to FireDock (as an input) to produce the top 10 refined structures of associated GalNAcT protein-ligand [40].

## 3. Results

### 3.1. SNP Analysis

The dbSNP-NCBI database search for nonsynonymous and synonymous SNPs located in exonic, intronic, and UTR regions of *GalNAc-T1* gene revealed a total of 891 SNPs of which the exonic region is comprised of 18 (2%) nsSNPs and 11 (1.2%) sSNPs, whereas intronic regions contained 836 (93%) SNPs and mRNA UTR region consisted of 26 (2.9%) SNPs. However, search for nonsense, frameshift, stopgain mutations did not show any results in NCBI-dbSNP. We selected nonsynonymous SNPs from the exonic region and UTR SNPs (5′ and 3′) from the intronic region for our analysis.

### 3.2. Identification of Functional SNPs in Coding Regions

Identification of functional SNPs was done by predicting those which substitute the amino acids that are critical for *GalNAc-T1* function. This *in silico* analysis was performed and validated using 4 different computational tools, namely, SIFT, PolyPhen-2, PANTHER, and SNPeffect.

#### 3.2.1. Analysis of Molecular Phenotypic Effects by SIFT

By using sequence homology, SIFT characterizes the effect of amino acid substitution on protein function. From SIFT results (Table 1), a total of 3 (16.6%) nsSNPs were predicted as damaging (score of 0.00–0.04) by SIFT, whereas the remaining 15 nsSNPs (83.33%) were simulated to be tolerated (score of 0.08–0.55). The 3 SNPs (rs113616262, rs34304568, and rs199977475) of *GalNAc-T1* gene are filtered to be functional by SIFT.

#### 3.2.2. Simulation of Functional Consequences by PolyPhen-2

For a given threshold of Naïve Bayes probabilistic score, PolyPhen-2 calculates the true positive rate as a fraction of correctly predicted mutations. From 18 nsSNPs of *GalNAc-T1 *gene, only 4 (22%) nsSNPs were predicted “probably damaging” (score of 0.96–1.00), whereas 14 (78%) were classified as benign (score of 0.411–0.00). The ranking of SNPs on the basis of PolyPhen-2 scores enables us to assess the potential-quantitative effect of SNPs on native protein (Table 2). PolyPhen-2 predicted functional 4 SNPs including “rs113616262, rs34304568, and rs199977475” and thereby validated the results predicted from SIFT.

#### 3.2.3. Functional Characterization by PANTHER and Fathmm

PANTHER characterizes likely functional effect of amino acid variation by means of HMM-based statistical modeling and evolutionary relationship. We performed PANTHER analysis of GalNAc-T1 nsSNPs in order to add another layer of refinement in SNPs characterization. A total of 4 SNPs possessed the subPSEC score less than −3 and were therefore classified as tolerated. The remaining 14 amino acid variants (D46G, P54L, D71E, D71H, V216 M, G258V, N379H, I384V, G401D, Y414D, N440H, and P510T) were found to be deleterious with subPSEC score in between −3 and −10 (Table 2). PANTHER-cSNP tool simulated rs113616262 and rs34304568 with the highest subPEC score of −7.4941 and −9.17274, respectively.

From Fathmm, only rs34304568 showed the damaging effect of amino acid substitution (Y414D) with −1.82 score.

#### 3.2.4. Functional Analysis by SNPeffect

SNPeffect database predicts the impact of SNPs on aggregation-prone regions, amyloid-forming regions, Hsp70 chaperone binding sites, and structural stability of human proteins. TANGO analysis showed that L25V variant increases (dTANGO score is 69.27), and Y414D decreases (dTANGO score is −154.10), the aggregation tendency of the GalNAc-T1 protein. However, WALTZ analysis revealed that Y414D (dWALTZ score is −209.48) also possessed the ability to decrease protein amyloid propensity. None of the variants were predicted to alter the chaperone binding sites for Hsp70 chaperones. When analyzed by FoldX, severe reduction in stability was demonstrated by S143P (7.50 Kcal/mol), G258V (7.82 Kcal/mol) Y414D (6.03 Kcal/mol) variants, while N440H (1.67 Kcal/mol), P510T (2.25 Kcal/mol) and N541S (0.55 Kcal/mol) were accounted to slightly reduce the stability of GalNAc-T1 protein.

#### 3.2.5. Characterization of Functional SNPs by Concordance Analysis

The efficacy of functional SNP prediction can be made more reliable by combining the results of empirical and support vector machine (SVM) based approaches. Hence, we performed the concordance analysis to get the integrated picture with SIFT, PolyPhen-2, PANTHER, and SNPeffect tools. Out of the 18 nsSNPs, 3 (16.66%) were predicted to be “deleterious” by SIFT, whereas this prediction rate was increased to 4 (22%) as “probably damaging” when analyzed by PolyPhen-2, 5 (27.77%) as “severely destabilizing” by SNP effect and 14 (78%) as “deleterious” by PANTHER. From concordance analysis, 3 amino acid variants (S143P, G258V and Y414D) were commonly predicted by all 4 of the *in silico* tools (SIFT, PolyPhen-2, PANTHER, and SNPeffect).

#### 3.2.6. Identification of Functional SNPs in Regulatory and Conserved Regions

Using empirical Bayesian inference, ConSurf database characterizes the evolutionary conservation of amino acids. Our ConSurf results showed that only S143 was located in highly conserved region and predicted to have functional impact on GalNAc-T1 protein. The remaining two nsSNPs responsible for G258V and Y414D are found in the vicinity of conserved residues and found to be buried in wild type residues by NetSurfP (Table 3).

FastSNP assigns the risk ranking for SNPs after excising functional effect information. From FastSNP results, we found 2 SNPs with rs72964406 (ESE motif: C*C*TCATG; score is 2.897886) and rs34304568 (ESE motif: G*G*ACCTAG; score is 2.088850), located in coding regions and altered ESE (exonic splicing enhancers) motifs indicating that all these nsSNPs may have the ability to affect the level, position, and timing of gene expression or regulate the splicing of *GalNAc-T1* gene transcript. No functional role was predicted for the SNPs, which are found be located in 3′ UTR region.

### 3.3. AMBER-Energy Minimization, Solvent Accessibility, and Electrostatic Interaction Effects of Functional SNPs on GalNAc-T1 Protein

**Figure 2:**
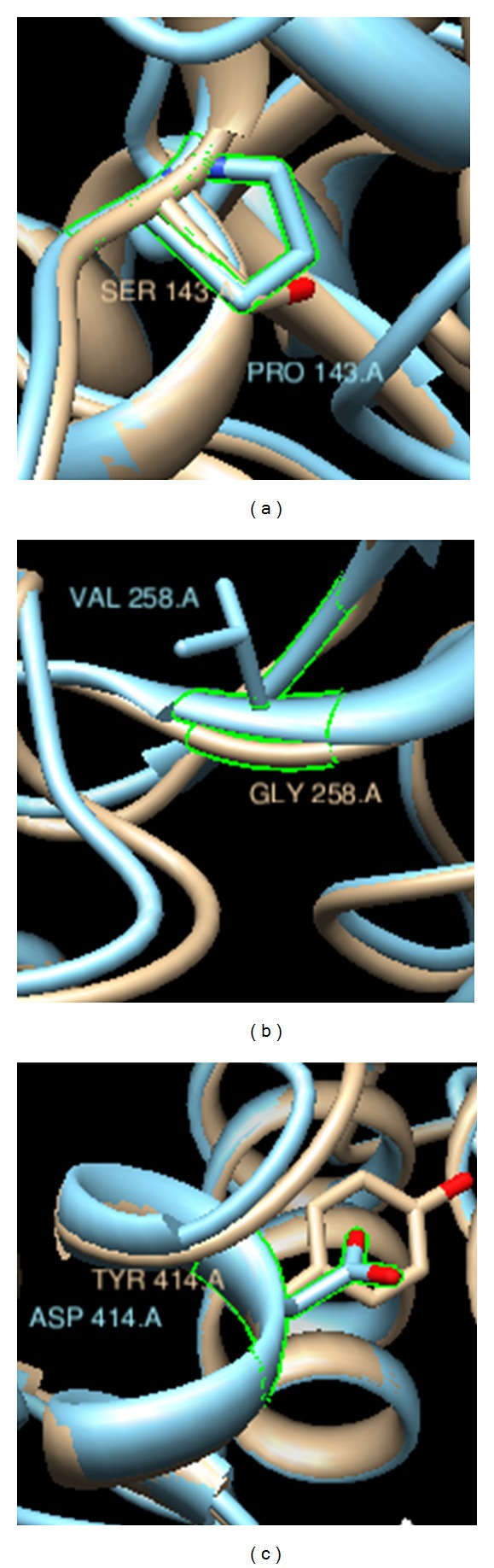
Superimposed structures of *GalNAc-T1* native (camel color) and mutant (blue color) models to visualize the stereochemical conformation of wild type and mutant residues at 143, 258, and 414 positions.

**Figure 3:**
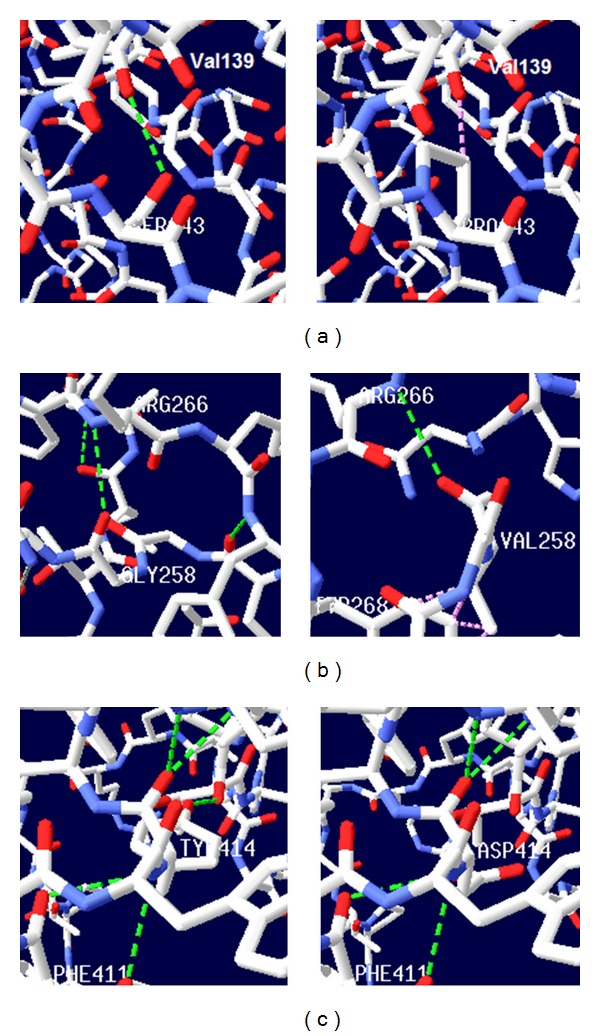
H-bonding (green discontinuous line) interactions and clashes (pink discontinuous line) of wild type and mutant analogues with the vicinal amino acid residues. (a) Ser143 is examined with single H-bonding with Val139 and converted into clash as a result of Pro at the same position. (b) At 258 position, one H-bond is observed with Arg266 in both native (Gly258) and mutant (Val258) structures, but a network of clashes appeared between Val258 and Tyr268. (c) Tyr414 is visualized with five H-bonding interactions for Pro410 and Asn417, and one H-bond is distorted due to appearance of mutant aspartic acid at the same position.

Based on multiple-threading alignments, I-TASSER builds high-quality 3D structures of protein molecules from their amino acid sequence. After retrieving protein FASTA sequence of *GalNAc-T1* from UniProt (ID “Q10472”) database, protein sequence was given to I-TASSER as an input. The I-TASSER tool created the 5 full-length models for GalNAc-T1 protein (with *C*-scores: −0.75, −1.29, −1.89, −2.71, and −2.83) by excising top 10 structures with *C*-scores after targeting the PDB library hits (Table 4). Among the 5 predicted models, model 1 carried the high-quality confidence in the form of *C*-score (−0.75), TM-Score (0.62 ± 0.14), and RMSD (9.3 ± 4.6Å), hence, it was selected for further analysis using Chimera, Swiss Pdb viewer, GROMOS96, and AMBER ff99SB software to visualize and compute the total energy before and after energy minimization. The superimposed structures of mutant and wild-type residues with their surface were visualized in Figure 2. The total energy of native structure was found to be 13294.691 kJ/mol before the energy minimization stage and it was −13882.539 kJ/mol after the energy minimization step, whereas mutant structures exhibited deviation in total energy value calculated before and after energy minimization (Table 5). Among 3 screened mutations, G258V showed the increase in total energy of *GalNAc-T1* before (5977791.00 kJ/mol) and after energy minimization (−10727.144 kJ/mol). Chimera and Swiss PDB viewer were used to visualize the structural features of amino acids in native and mutant protein chains. During structural visualization for all 3 mutations, only mutant residue (valine) at 258 position showed a network of clashes with Tyr268 (Figure 3). Additionally, S143P, G258V, and Y414D variants were analyzed for solvent accessibility (in form of *Z*-score) and stability, and a decrease in both parameters was observed for all three variants (Table 3).

### 3.4. The Structural Impacts of Functional *GalNAc-T1* Mutations

**Figure 4:**
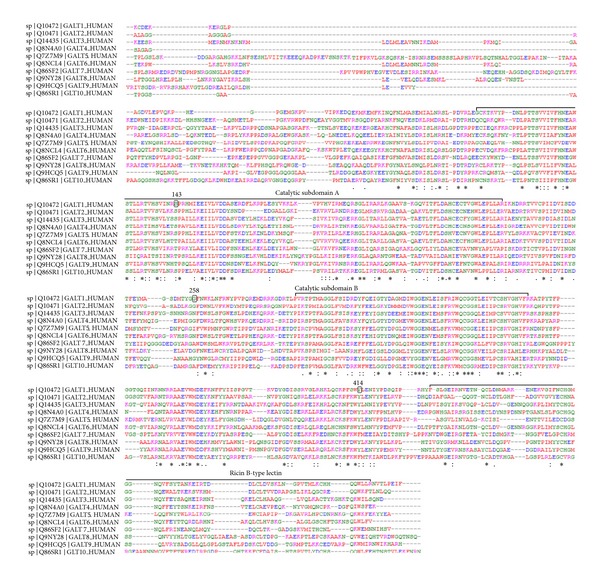
Multiple sequence alignments and evolutionary conservation behaviour among human *GalNAc-T1* to *GalNAc-T10* members of GALNTs family. S143 and Y414 residues are found conserved in the catalytic subdomain A and linker B domain (area between catalytic subdomain B and Ricin B-type lectin), respectively. G258 is observed in the vicinity of conservation groups with strongly similar properties (shown with “:”).

Project HOPE simulates the structural features of amino acid substitution on native protein structure. The S143 residue is located within a stretch of residues annotated in Uniprot as catalytic domain A (Figure 4 from Clustal Omega). The hydrophobicity and size difference between wild-type and mutant residue (Pro) can disrupt the H-bonding interactions with the neighbouring residues, and hence the protein framework. The wild-type residue forms a hydrogen bond with the valine on position 139. Due to high rigidity of proline moiety, this mutation might abolish the required flexibility of the protein at this position and could affect the catalytic tendency. Based on conservation behaviour, this mutation is probably damaging to the protein.

For G258V, the wild-type residue is resided in the linker region between catalytic subdomains A and B and can distract the required flexibility of core structure by substituting Gly258 with valine. Additionally, the structural analysis of Val258 showed some clashes for Tyr268 which may contribute to the extra energy in the protein structure, and hence the decrease in stability.

In the Y414D variant, Tyr414 indicated five H-interactions with Pro410, Phe411, and Asn417, whereas mutant Asp414 exhibited the four H-bonding interactions in the core of the protein (or protein complex) due to the differences in charge density and hydrophobicity between wild-type and mutant residue.

### 3.5. Simulation for *GalNAc-T1* Molecular Binding Sites and Protein-Protein Interactions

Including the structure-based prediction of protein function, FTSite identifies the ligand binding sites for a particular protein. Upon querying with FTSite, a considerable difference in the number of ligand binding sites was observed between wild type and mutant forms (S143P, G258V, and Y414D) of GalNAc-T1 protein (Table 6).

**Figure 5:**
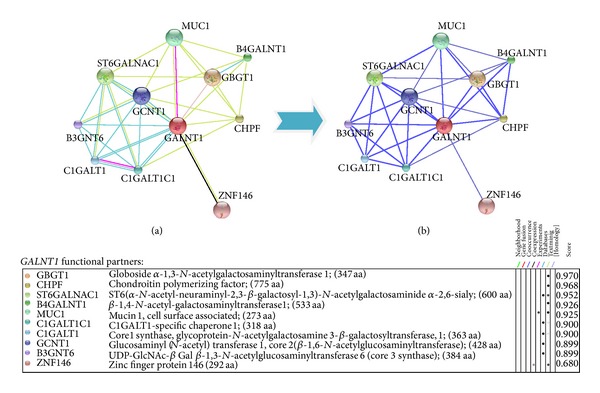
GalNAc-T1 protein-protein interactions with 09 partners. One colour is given to each type of evidence in the predicted functional links (edges) among eight coloured lines. (a) From experimental basis (with score 0.925), only *MUC1 *is observed for interaction with *GalNAc-T1*. From text-mining data, *GalNAc-T1* interactions are observed for *GBGT1, CHPF, ST6GALNAC1, B4GALNT1,* and *MUC1* proteins with 0.970, 0.968, 0.952, 0.926, and 0.925 scores, respectively. The remaining interactions with *C1GALT1C1, CIGALT1, GCNT1,* and *B3GNT6* proteins are simulated with STRING score ranging from 0.900 to 0.899. (b) Strong association pattern (thick blue lines) of *GalNAc-T1* is predicted for *GBGT1, CHPF, ST6GALNAC1, B4GALNT1, MUC1, C1GALT1C1, C1GALT1, GCNT1*, *B3GNT6,* and *ZNF146* partners with high confidence. For *ST6GALNAC1-GBGT1, ST6GALNAC1-MUC1, GCNT1-CHPF, B4GALNT1-GCNT1, CHPF-C1GALT1C1,* and *GALNT1-ZNF146* pairs, weak associations are examined in the form of thin blue lines.

**Figure 6:**
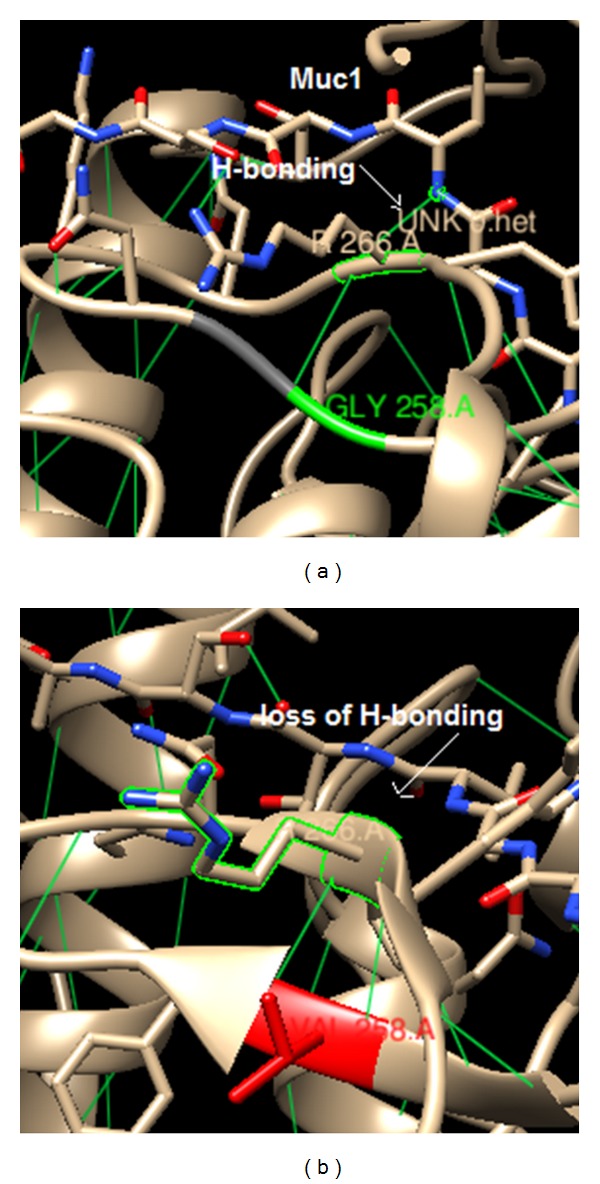
3D visualization of G258V variant with *MUC1*. (a) Ligand binding of *GalNAc-T1* native structure with *MUC1* indicated single H-bonding interaction (green line) of Gly258 residue (green portion) with Arg266 which is further connected with the GVTSA (tandem repeat region of *MUC1*) by means of H-bonding interaction. (b) Picturing of GalNAc-T1 mutant protein structure is observed with one H-bonding loss between Arg266 residue and the *MUC1* tandem repeat motif.

STRING database annotates the functional interactions among the proteins, in a cell. STRING results predicted the functional association pattern of GalNAc-T1 protein (physical and functional) with MUC1, GBGT1, CHPF, ST6GALNAC1, B4GalNAc-T1, C1GALT1C1, C1GALT1, GCNT1, and B3GNT6 partners with high confidence, shown with bold lines in Figure 5. Based on the confidence scores of GalNAc-T1 protein interactions, MUC1, a densely *O*-glycosylated transmembrane protein critical to cellular integrity, was chosen for molecular docking analysis in order to identify the plausible structural and functional implications of S143P, G258V, and Y414D variants. The H-bonding interaction existing between Gly258 (green portion) and Arg266 residues of native GalNAc-T1 is in turn critical for its interaction with N-H of Thr, located in GVTSA (tandem repeat region) of *MUC1* by means of H-bonding. However, due to the replacement of Gly at 258 position with Val in GalNAc-T1, the H-bonding interaction with *MUC1* was distorted (Figure 6).

After STRING, mucin biosynthetic pathway was retrieved from KEGG database to retrieve the molecular reaction and interaction behaviour for* GalNAc-T1* and other partner enzymes (*ST6GALNAC1, B4GALNT1, C1GALT1C1, C1GALT1, GCNT1*, and *B3GNT6*), to corroborate the STRING results.

## 4. Discussion

The human *GalNAc-T1* gene is located on chromosome 18q12.1 region with 11 exons (58.82 kb) [1]. A 3,847 bp length m-RNA encodes the functional GalNAc-T1 protein with 559 amino acids (64.2 kDa, pI 7.4). However, alternative splicing of mRNA yields different polynucleotide chain lengths, that is, 1,770 bps (499 aa) and 1120 bps (105 aa) amino acids, respectively. So far, approximately 900 variants located in noncoding, coding, and regulatory regions of human *GalNAc-T1* gene are described in dbSNP database to date. With the advent of high throughput (whole exome and genome) sequencing practices, the number of genetic variations is growing day by day in an efficient manner [18, 24, 41, 42]. Hence, an important task of human genetics lies in delineating those amino acid variants which can impose specific structural and functional consequences on protein function [41].

In the current investigation, screening for functional *GalNAc-T1* genetic variants in coding region was performed using sequence- and structure-based algorithms such as SIFT, PolyPhen-2, PANTHER, and SNP effect. From functional analysis of SNPs, SIFT predicted that 17% of total nsSNPs are of deleterious type, whereas PolyPhen-2 predicted ~22% of total nsSNPs to be highly deleterious. But common predictions of SIFT and PolyPhen-2 showed that 16% of variants were functional. The significant difference in outputs of SIFT and PolyPhen-2 algorithms is most likely due to utilization of different protein sequence alignments, used to characterize the variants [42]. However, good coherence and accuracy between PANTHER and SIFT (use of similar scoring matrices) have predicted 4 (22.22%) nsSNPs of *GalNAc-T1* to be functional. SNPeffect predicted that 3 (16.6%) nsSNPs of the variants to be highly destabilizing. The accuracy of this prediction percentage remained the same even when combined with PolyPhen-2 analysis but increased to 6 (33.33%) nsSNPs with PANTHER. By comparing the output of all the 4 different *in silico* tools (SIFT, PolyPhen-2, PANTHER-cSNP, and SNPeffect), nsSNPs that encodes S143P, G258V, and Y414D variants were found to be functionally significant. The differences in prediction capabilities can be attributed to the fact that every method uses different sets of sequences and alignments. When compared, sequence-based prediction analysis has a number of advantages over structure-based analysis, due to the fact that it considers all types of effects at the protein level and is well suitable for proteins with known relatives. But sequence-based predictions are unable to explain the underlying mechanisms between genotype and phenotype relationships for most of the proteins. On the contrary, the structure-based approach has limitations in that it cannot be implemented for proteins with unknown 3D structures. *In silico* investigation tools that integrate both sequence- and structure-based approaches will be of added advantage in providing reliable prediction results with wider coverage of different aspects of SNP analysis. However, variability in prediction outputs of these algorithms reflects both advantages and disadvantages. Therefore, decisions regarding the selection of a suitable tool for SNP analysis must be subjective to the specific objectives of the investigation undertaken.

Our ConSurf and Clustal Omega results indicated that nsSNPs at positions S143P and Y414D were found in the highly conserved region and predicted to have potential impact on GalNAc-T1 protein. The third mutation (G258) is observed in the vicinity of conservation groups with strongly similar properties, shown by “:” in Figure 4. Besides that, the SNPs which encode functional polypeptides are those regulatory SNPs that control the gene expression. FastSNP tool helped us to prioritize the deleterious SNPs, based upon their impact on determining protein structure, deviance in transcriptional levels of the sequence, modification in the premature translation termination, and aberrations in the positions at promoter region for transcription factor binding. Altered *O*-glycosylation machinery due to aberrant *GalNAc-T1* expression may result in aberrantly glycosylated proteins and new antigenic targets, thereby altering host immunogenic response in ovarian cells [43, 44]. The two SNPs i-e rs72964406 (ESE motif: C*C*TCATG; score is 2.897886) and rs34304568 (ESE motif: G*G*ACCTAG; score 2.088850) were located in coding regions and altered ESE (exonic splicing enhancers) motifs indicating that all these nsSNPs may have the ability to affect the level, location, and timing of gene expression or regulate the splicing of *GalNAc-T1* gene transcript.

The GalNAc-T1 protein structure carries an *N*-terminal transmembrane domain, a stem region, a lumenal catalytic domain (catalytic A/GT1 domain and catalytic B/GalNAc-T domain), and a C-terminal ricin-type lectin region (Figure 4). Although all GalNAc-transferases are reported to share common structural features and the conserved motifs described above, the exact role of each domain in catalysis remains unknown. Several biological roles have been described for GalNAc-T1; for example, loss of GalNAc-T1 leads to reduced leukocyte recruitment and increased rolling velocity in vivo, suggesting the predominant role for GalNAc-T1 in attaching functionally relevant *O*-linked glycans to selectin ligands [45]. Protein structural analysis is performed to fine-tune the picture, drawn by the concordance analysis of SIFT, PolyPhen-2, and PANTHER. Calculation of solvent accessibility and force field energy have provided an insight into the structural and functional impacts of amino acid substitutions. 3D models are constructed to visualize deviation among the mutant and wild-type protein models. In S143P, the required protein framework (hydrophobicity and H-bonding interactions) seems to be disturbed at this position and could be damaging for the native structure. The G258V substitution carries the tendency to disturb the local structure by reframing the backbone flexibility. Y414D variant is found to be associated with regulation of protein misfolding. Due to high difference in total energy for native and mutant (carrying G258V variant) models, the proper functioning of *GalNAc-T1 *can be disturbed. The analysis of sequence-based motifs and conserved residues is a reliable method to identify the functional amino acid residues that are important for binding, catalysis, and stability of proteins. Upon analysing with FTSite, a difference in the ligand binding sites is observed between wild type (20 binding site) and mutant forms (S143P: 18 binding sites; G258V: 23 binding sites; Y414D: 23 binding sites) of GalNAc-T1 protein.

**Figure 7:**
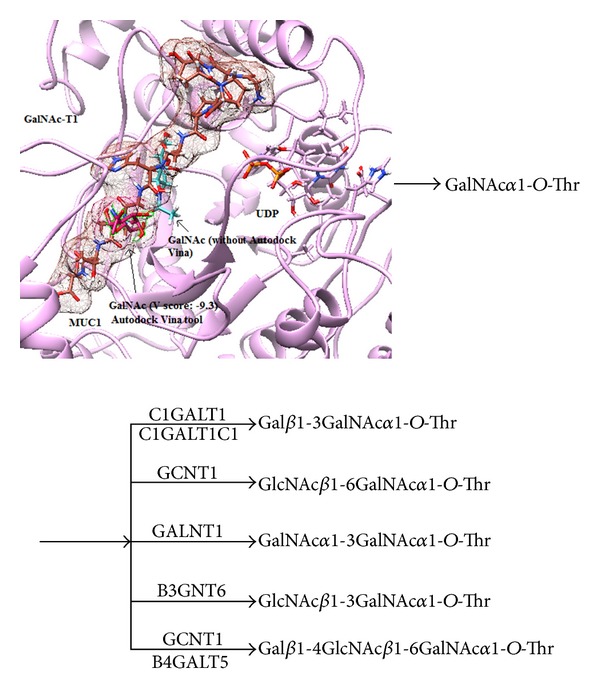
Complex structure of *GalNAc-T1* enzyme with UDP, *MUC1* (tandem repeat region “GSTAPPAHGVTSAP”), and GalNAc residue. GalNAc sugar is attached at Thr of tandem repeat region in *MUC1*. His125 and Asp156 residues from catalytic A domain and Ile315, Trp316, and Thr350 from catalytic B domain are found in contact with UDP (orange red color). Under the action of *ST6GALNAC1, B4GALNT1, C1GALT1C1, C1GALT1, GCNT1,* and *B3GNT6,* GalNAc1-*O*-Thr is stereospecifically converted into different glycan chains to regulate the mucin biosynthesis pathway.

Protein-protein interaction analysis is a comprehensive way to understand the global organization of proteomes in the context of a functional network. Interaction network displays biomolecules as nodes and interactions connecting the two nodes as edges. Currently, functional network view for a single genome is widely used to improve the statistical power in human molecular genetics [39], to aid drug discovery, to better understand metabolic pathways, and to derive genotype-phenotype correlations [18, 46]. STRING maps have shown that *GalNAc-T1* interacts with GBGT1, CHPF, ST6GALNAC1, B4GALNAC-T1, MUC1, C1GALT1C1, C1GALT1, GCNT1, B3GNT6, and ZNF146 partners by 36 interactions. The Strong interaction patterns were observed for GBGT1, CHPF, ST6GALNAC1, B4GALNT1, MUC1, C1GALT1C1, C1GALT1, GCNT1, B3GNT6, and ZNF146 partners. From KEGG-Pathway analysis, biochemical (stereospecific) reactions for GalNAc-T1, ST6GALNAC1, B4GALNT1, MUC1, C1GALT1C1, C1GALT1, and GCNT1 indicated the individual and combined role of each enzyme in regulation of glycosylation phenomena to carry out mucin biosynthesis pathway (Figure 7). Noticeably, most of these enzymatic proteins are involved in cell adhesion and membrane transport. Therefore, aberrant glycosylations in the form of mutations in *GalNAc-T1* gene could distort the mucin and many associated pathways (ERK, SRC, and NF-kappa-B pathways) involved in maintaining cellular response and integrity. Of the 3 *GalNAc-T1* variants tested, G258V was found to disturb the interaction of Arg266 of *GalNAc-T1* with N-H of Thr (in GVTSA; tandem repeat region) of *MUC1* [47]; thus, it might impair the possible transfer of GalNAc residue from UDP-GalNAc to hydroxyl group of Ser/Thr, during *O*-glycosylation reaction (Figure 7). Over expression, aberrant intracellular localization, and changes in glycosylation of *MUC1* and *MUC7* are often reported in tumors of colon, breast, ovarian, lung, and pancreatic origin [24, 48]. Since *MUC1* plays an essential role in cellular signalling and microbial pathogenicity, it is conceivable to expect that G258V mutation is pathogenic to cellular integrity and susceptibility to certain human disease.

## 5. Conclusion

This study represents the first comprehensive investigation that identified the functional SNPs in *GalNAc-T1* gene using sequence- and structure-based homology algorithms. Although there were notable differences in the prediction basis of selected *in silico* algorithms, our concordance analysis corroborated the characterization of suspected SNPs that could play significant roles in cellular biology. We defined the structural consequences of S143P, G258V, and Y414D variants on GalNAc-T1 protein in the form of solvent accessibility, electrostatic interaction, energy calculation, and multiple alignment conservation. From potential SNPs, the G258V mutation is predicted to cause considerable change in total energy, electrostatic intramolecular interactions, and functional interaction behaviour of GalNAc-T1 protein with *MUC1* protein and thereby may impact the possible transfer of GalNAc residue from UDP-GalNAc to hydroxyl group of Ser/Thr during *O*-glycosylation of proteins. Additionally, 2 regulatory SNPs that influence the splicing and expression pattern of *GalNAc-T1 *gene have also been identified. Altered *GalNAc-T1* function due to genetic variations and mRNA expression might play a critical role in determining susceptibility to complex diseases. Furthermore, protein-protein interaction pathway and protein-ligand docking have helped us to understand the stereochemical (anomery and linkage) roles of *GalNAc-T1* and associated enzymes in mucin biosynthetic pathway. Finally, the *in silico* based nsSNP predictions would not just be of use in deriving genotype-phenotype relations but to some extent explain the molecular basis for varied interindividual response to certain drugs.

## Figures and Tables

**Table 1:** List of nsSNPs with rs IDs found to be functionally significant by SIFT Tool.

rs IDs	Allele change	Amino acid change	SIFT score	SIFT prediction	Median
rs143502430	c.13G>A	A5T	0.55	TOLERATED	2.95
rs141224997	c.58T>G	L20V	1	TOLERATED	2.66
rs138822379	c.73C>G	L25V	0.07	TOLERATED	2.67
rs199595808	c.137A>G	D46G	0.3	TOLERATED	2.51
rs72964406	c.161C>T	P54L	0.28	TOLERATED	2.51
rs139185162	c.213T>G	D71E	1	TOLERATED	2.38
rs150799030	c.211G>C	D71H	0.07	TOLERATED	2.38
rs113616262	c.427T>C	**S143P**	0	**DAMAGING**	2.37
rs201338228	c.646G>A	V216M	0.14	TOLERATED	2.37
rs199977475	c.773G>T	**G258V**	0.04	**DAMAGING**	2.37
rs144282744	c.1135A>C	N379H	0.1	TOLERATED	2.37
rs146565032	c.1150A>G	I384V	0.52	TOLERATED	2.38
rs142387342	c.1202G>A	G401D	0.34	TOLERATED	2.37
rs34304568	c.1240T>C	**Y414D**	0	**DAMAGING**	2.37
rs151329739	c.1318A>C	N440H	0.08	TOLERATED	2.37
rs142110831	c.1528C>A	P510T	0.16	TOLERATED	2.37
rs145884536	c.1622A>G	N541S	0.45	TOLERATED	2.38
rs200444543	c.1657G>A	V553I	0.39	TOLERATED	2.66

SIFT score ≤ 0.05: damaging.

**Table 2:** Characterization of SNPs by using PolyPhen-2, PANTHER, and Fathmm based classification systems.

Amino acid change	PolyPhen-2			PANTHER		Fathmm	
A1#A2	Score	Prediction	Specificity/sensitivity	subPSEC Score	Prediction	Score	Prediction
A5T	0.411	Benign	0.90/0.89	−2.96529	Tolerated	0.59	Tolerated
L20V	0.000	Benign	0.00/1.00	−2.65438	Tolerated	0.69	Tolerated
L25V	0.960	Probably damaging	0.95/0.78	−3.45127	Deleterious	0.35	Tolerated
D46G	0.004	Benign	0.59/0.97	−3.34504	Deleterious	0.51	Tolerated
P54L	0.001	Benign	0.15/0.99	−4.27568	Deleterious	0.56	Tolerated
D71E	0.000	Benign	0.00/1.00	−3.40972	Deleterious	0.68	Tolerated
D71H	0.132	Benign	0.86/0.93	−5.24027	Deleterious	0.49	Tolerated
**S143P**	**0.999**	**Probably damaging**	**0.99/0.14**	**−7.4941**	**Deleterious**	0.15	Tolerated
V216M	0.411	Benign	0.90/0.89	−5.56762	Deleterious	0.26	Tolerated
**G258V**	**1.000**	**Probably damaging**	**1.00/0.00**	**−4.40932**	**Deleterious**	0.35	Tolerated
N379H	0.000	Benign	0.00/1.00	−3.41433	Deleterious	−0.35	Tolerated
I384V	0.000	Benign	0.00/1.00	−3.81526	Deleterious	−0.31	Tolerated
G401D	0.000	Benign	0.00/1.00	−3.23851	Deleterious	1.56	Tolerated
**Y414D**	**1.000**	**Probably damaging**	**1.00/0.00**	**−9.17274**	**Deleterious**	−1.82	**Damaging**
N440H	0.048	Benign	0.83/0.94	−4.06922	Deleterious	−1.17	Tolerated
P510T	0.009	Benign	0.77/0.96	−3.46487	Deleterious	−1.09	Tolerated
N541S	0.000	Benign	0.00/1.00	−2.80107	Tolerated	−1.03	Tolerated
V553I	0.000	Benign	0.00/1.00	−2.409	Tolerated	−0.98	Tolerated

PolyPhen-2: probably damaging (probabilistic score > 0.85), possibly damaging (probabilistic score > 0.15), and benign (remaining).

PANTHER: deleterious or intolerant (subPSEC score ≤ −3), less deleterious (subPSEC score ≥ −3).

**Table 3:** Surface accessibility of wild-type and mutant variants in *GalNAc-T1*.

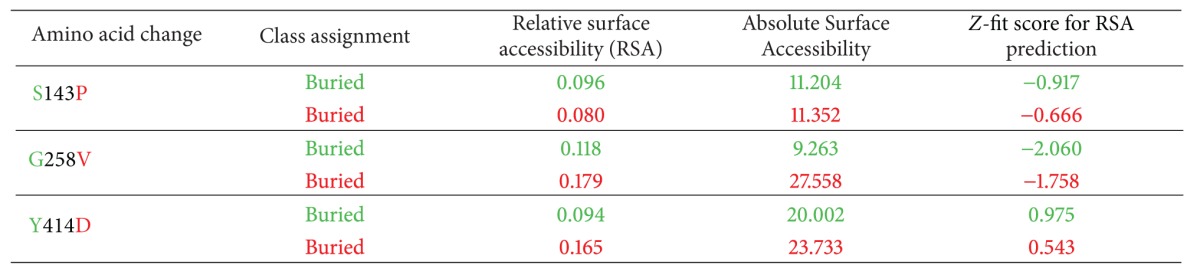

**Table 4:** Top 10 templates used by I-TASSER to create the high-quality models for human *GALNT1* secondary structure.

Rank	PDB Hit	Iden1	Iden2	Cov.	Norm. *Z*-score
1	2ffuA	0.45	0.41	0.88	3.12
2	2d7iA	0.43	0.42	0.91	7.15
3	2ffuA	0.45	0.41	0.88	8.16
4	2ffuA	0.45	0.41	0.88	5.97
5	2ffuA	0.45	0.41	0.88	4.41
6	2d7iA	0.43	0.42	0.91	7.11
7	2ffuA	0.45	0.41	0.88	7.99
8	2d7rA	0.43	0.42	0.92	6.42
9	1xhbA	0.99	0.79	0.80	3.06
10	2ffuA	0.45	0.41	0.88	6.21

Rank of templates represents the top ten threading templates used by I-TASSER. “Ident1” is the percentage sequence identity of the templates in the threading aligned region with the query sequence. “Ident2” is the percentage sequence identity of the whole template chains with query sequence. “Cov” represents the coverage of the threading alignment and is equal to the number of aligned residues divided by the length of query protein. “Norm. *Z*-score” is the normalized *Z*-score of the threading alignments. Alignment with a Normalized *Z*-score > 1 mean a good alignment and vice versa. The top 10 alignments reported above (in order of their ranking) are taken from the following threading programs: 1: MUSTER 2: HHSEARCH 3: SP3 4: PROSPECT2 5: PPA-I 6: HHSEARCH I 7: SPARKS 8: SAM T99 9: MUSTER 10: HHSEARCH.

**Table 5:** Total energy of native and mutant structures before and after energy minimization.

Amino acid variants	Total energy before energy minimization (kJ/mol)	Total energy after energy minimization (kJ/mol)
Native	13140.137	−13882.539
S**143**P	13392.757	−13767.374
G**258**V	59777.910	−10727.144
Y**414**D	12995.687	−13918.198

**Table 6:** Ligand binding sites for wild type and mutant forms (S143P, G258V, and Y414D).

Wild type	S143P	G258V	Y414D
Asn82	Asn82	Asn82	Asn82
Phe84	Val123	Phe84	Phe84
Val123	Phe124	Val123	Val123
Phe124	His125	Phe124	Phe124
His125	Asn126	His125	His125
Asn126	Glu127	Asn126	Asn126
Glu127	Ala128	Glu127	Glu127
Thr131	Lau132	Asp156	Asp156
Asp156	Asp156	Gly188	Leu189
Gly188	Leu189	Leu189	Asp209
Leu189	Asp209	Asp209	Ala210
Arg193	Ala210	Ala210	His211
Asp209	His211	His211	Ile315
Ala210	Ile315	Ile315	Trp316
His211	Trp316	Trp316	His344
Ile315	Arg347	His344	Val345
Trp316	Thr350	Val345	Phe346
His344	Pro351	Phe346	Arg347
Val345	—	Arg347	Lys348
Phe346	—	Lys348	Ala349
**—**	—	Ala349	Thr350
**—**	—	Thr350	Pro351
**—**	—	Pro351	Tyr352
